# Genomic surveillance of avian-origin influenza A viruses causing human disease

**DOI:** 10.1186/s13073-018-0560-3

**Published:** 2018-06-27

**Authors:** Tommy T. Lam, Oliver G. Pybus

**Affiliations:** 1 State Key Laboratory of Emerging Infectious Diseases, School of Public Health, University of Hong Kong, Hong Kong, SAR China; 2 Department of Zoology, University of Oxford, South Parks Road, Oxford, OX1 3PS UK

**Keywords:** Influenza, Genomics, Epidemiology, Transmission, Birds, Pathogenicity

## Abstract

Avian influenza A viruses (AIVs) pose a threat to global health because of their sporadic zoonotic transmission and potential to cause pandemics. Genomic surveillance of AIVs has become a powerful, cost-effective approach for studying virus transmission, evolution, and dissemination, and has the potential to inform outbreak control efforts and policies.

## Avian influenza A viruses and human disease

Influenza A viruses are a persistent and significant threat to public health. In addition to the recurring seasonal epidemics caused by human influenza A viruses, infections may be caused by influenza viruses from animals, notably birds and swine. Only occasionally does an influenza A virus of animal origin cause human infections that develop into a global pandemic (most recently in 2009); the great majority of zoonotic influenza A infections do not transmit among humans. Despite this, investigation of avian influenza viruses (AIV) is crucial, not only because of the sporadic human infections they cause but also because they are a potential source of future influenza pandemics, against which human populations have less immunity.

Influenza A viruses are classified into subtypes according to their two surface proteins, hemagglutinin (H) and neuraminidase (N). Annual influenza A epidemics in humans are caused by only two subtypes, H3N2 and H1N1, and the 2017–18 influenza epidemic season was more severe than average in the US, Europe, and Australia. By contrast, birds are a natural reservoir of influenza A viruses and maintain a much greater diversity of different subtypes. A number of AIV strains have proven fatal in a small proportion of the people they infect, most of whom have had direct contact with poultry. The best known AIV strain is the highly pathogenic Asian H5N1 lineage, which was first detected in humans in 1997 [1]. This lineage has since infected at least 860 people worldwide, approximately half of whom died, and continues to circulate and evolve in bird populations. More recently, in February 2013, a novel H7N9 AIV emerged [1]. As of February 2018, this virus and its descendants have caused 1567 infections and 615 deaths among people in China. Although no H7N9 human cases have been reported since that date, it remains to be seen whether human infections of this virus will re-emerge in the future.

## Genomic surveillance and epidemiology of AIVs

**Fig. 1:**
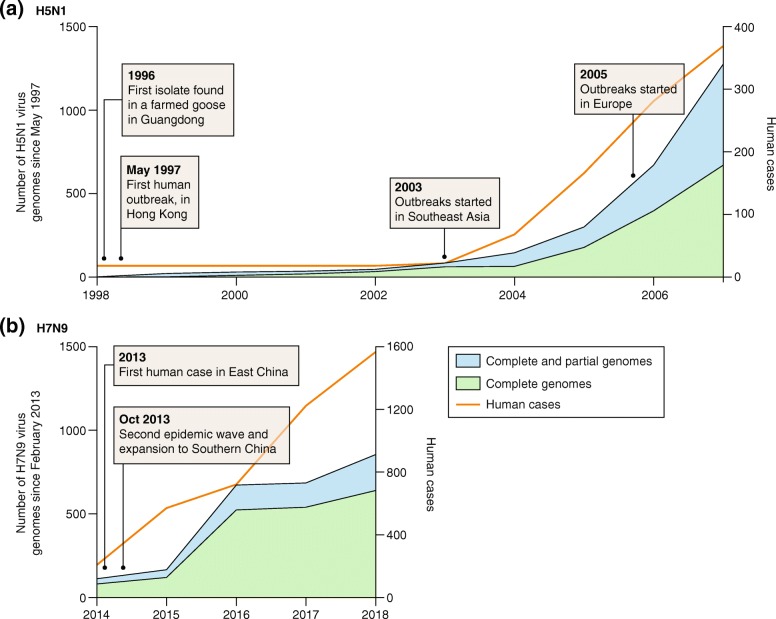
Cumulative numbers of H5N1 and H7N9 avian influenza virus genomes available in public databases since their first emergence dates in 1997 and 2013, respectively. Numbers of genomes (*left-hand y-axis*) are shown for both complete and partial genomes of **a** H5N1 and **b** H7N9. The *orange line* shows the cumulative number of human cases of each strain observed over the same timescale (*right-hand y-axis*). Selected events during the timeline of virus emergence and spread are indicated

Genomic epidemiology is playing an increasingly important role in the surveillance of avian influenza A viruses that cause sporadic zoonotic disease and which may represent a potential cause of future influenza pandemics. Virus genome sequences can be analyzed to track the transmission and evolution of outbreaks, and technological advances have allowed a continual rise in the speed and affordability of whole genome sequencing of the pathogens. A comparison of H5N1 and H7N9 genomics serves to illustrate the progress of genomic epidemiology over the past 20 years (Fig. 1).

In May 1997, a highly pathogenic H5N1 AIV emerged and killed a child, and subsequently caused six deaths among 18 infected people in Hong Kong that year. The first genome of that strain was published over 7 months later. By contrast, genomes representing the first few human cases of H7N9 infection in China in February 2013 were available in a public database within 1 month of their isolation. Notably, the number of published complete H7N9 genomes sequenced in the first year after its emergence was greater than the number of H5N1 genomes generated after 7 years of sequencing effort. Contemporary sequencing capacity is now sufficiently large that data generation is more likely to be affected by logistical, administrative, or bioinformatic constraints than by genomic ones. Furthermore, virus sequencing methods continue to advance, as illustrated by the recent report of the direct RNA sequencing of influenza A virus genomes [2]. Our increased ability to sequence influenza virus genomes means that evolutionary, genetic, and epidemiological insights can be gleaned more rapidly, and with greater detail, following the discovery of a new strain.

For example, virus genomic surveillance in animals undertaken after the discovery of H7N9 in 2013 in China confirmed that chickens were the main source of human H7N9 infection, and helped researchers to reconstruct the evolutionary origin of the virus [1]. The work revealed that H7N9 AIVs probably originated in wild waterbirds and were transmitted to domestic ducks, and from there to chickens, from which they acquired new internal genes (that is, influenza genes other than those coding for proteins H and N) from the H9N2 AIV lineage that is mostly maintained in chickens. The new H7N9 strain then spread rapidly in China, resulting in spill-over to human populations. The acquisition of H9N2 internal genes does not appear to be a random event, as some other AIV strains that have the potential to infect humans and were discovered in Chinese chickens around the same time (H7N7 and H10N8 [1]) also acquired these genes. Subsequent genomic studies of later H7N9 outbreaks have shown the virus to be widely disseminated throughout China and increasingly genetically diverse, as a result of viral mutation and reassortment between H7N9 and H9N2 viruses that co-circulated in live poultry markets [3]. These and other findings have helped to elucidate the central role of domestic ducks and chickens in driving zoonotic AIV infections in humans.

## Implications for disease control

Epidemiological surveillance typically focuses on detecting disease cases in a given region. These traditional studies can be complemented by phylogenetic analyses that combine virus genomes from different locations to gain insights into virus dissemination at larger spatial scales. Such methods have shown how H7N9 AIV spread from eastern to southern China, possibly as a result of poultry trading, from where it seeded many infections in the second epidemic wave [3]. Genomic epidemiology has been also used to evaluate the outcome of local interventions, such as the closure of live poultry markets, in controlling AIVs in a specific region [4]. When applied on a global scale, phylogeographic analyses revealed an association between long-distance bird migration and the spread to Europe and America in 2014 of the highly pathogenic Asian H5 subtype AIV [5], which cost the US poultry industry hundreds of millions of dollars.

In addition to supporting epidemiological studies, rapid virus genome sequencing can identify molecular markers that are associated with important influenza A virus phenotypes, and can thereby help to predict the pathogenicity, transmissibility, antigenicity, and drug sensitivity of newly emergent strains [6, 7]. Sequence-based assessment is now a routine component of many influenza surveillance programs and can inform estimates of emergence risk and help to evaluate the effectiveness of vaccines. Evolutionary analysis of influenza virus genomes is already being used to predict the antigenic evolution of the virus and, in collaboration with the World Health Organisation (WHO), is helping to inform influenza vaccine strain selection [8]. Furthermore, genomic surveillance showed that influenza viruses that were circulating during the 2016–17 season carried an N-linked glycosylation site that was absent from egg-adapted vaccines, reducing the effectiveness of those vaccines in antibody-binding experiments [9]. In some instances, the association between genome sequence and phenotype may be relatively straightforward, such as the presence of a polybasic cleavage site in the hemagglutinin connecting peptides, which in most instances confers high pathogenicity to AIV strains. For example, genome analysis of recent H7N9 viruses revealed mutations conferring high pathogenicity to birds and humans, highlighting the threats posed by AIV to public health and food supply [10]. In other cases, influenza virus mutations on different genes may interact, in which case a complete genome sequence is needed to forecast the viral phenotype in question. Despite their obvious importance, our understanding of the phenotypic effects of most influenza virus mutations is still poor. New ‘deep mutational scanning’ approaches that measure the effects of all possible viral mutations on growth in cell culture provide a promising way forward [11].

## Conclusions and future directions

Genomic surveillance is enabling the rapid investigation of the evolutionary and transmission dynamics of influenza viruses at local, regional, and international scales. In addition, viral genomes can be used to assist public health policies, such as live poultry market closures or the annual update of influenza vaccine strains.

Future interdisciplinary work that aims to combine virus genomes with data on human demography, international travel, wild bird movements, poultry trade, and human genetics therefore has great potential to improve our ability to predict the risk of influenza infection in people and poultry. Successful control of AIVs on a global scale will require increased genomic surveillance in poorly characterized regions, timely data sharing, and the development of new analytical methods to test hypotheses concerning influenza virus emergence and transmission. These aims can be achieved by close collaboration and coordination among countries, with the support of WHO, the Food and Agriculture Organization of the United Nations (FAO), the World Organisation for Animal Health (OIE), and other international health organizations.

## Abbreviations

AIV: Avian influenza A virus
H: Hemagglutinin
N: Neuraminidase
